# Prospective case–control study on pain intensity after the use of promethazine in patients undergoing videothoracoscopy

**DOI:** 10.3389/fmed.2024.1453694

**Published:** 2024-09-16

**Authors:** Xiangwei Zhou, Benhui He, Xia Zheng, Chao Li, Zeyu Mi, Mingqing Peng, Min Li

**Affiliations:** ^1^Department of Anesthesiology and Perioperative Medicine, The Affiliated Yongchuan Hospital of Chongqing Medical University, Chongqing, China; ^2^Department of Thoracic and Cardiovascular Surgery, The Affiliated Yongchuan Hospital of Chongqing Medical University, Chongqing, China

**Keywords:** promethazine, sufentanil, video-assisted thoracoscopic surgery (VATS), patient-controlled intravenous analgesia, thoracic surgery

## Abstract

**Objective:**

Effective and secure pain management following video-assisted thoracoscopic surgery (VATS) is crucial for rapid postoperative recovery. This study evaluated analgesic and sedative effects of sufentanil and promethazine in patient-controlled intravenous analgesia (PCIA) post-thoracic surgery, along with potential adverse reactions.

**Methods:**

In this prospective, randomized, controlled, double-blind, clinical study, 60 patients (American Society of Anesthesiologists status I–III) undergoing VATS were enrolled. The patients were randomized into experimental (Group P) or control (Group C) groups. PCIA was administered post-general anesthesia using a double-blind method. Group P received sufentanil (3 μg/kg) + promethazine (1 mg/kg) + 0.9% sodium chloride solution (100 mL total), while Group C received sufentanil (3 μg/kg) + 0.9% sodium chloride solution (100 mL total). PCIA settings included a 1-mL bolus and 15-min locking time. The primary outcomes were the visual analog scale (VAS) at rest and during coughing and sedation (Ramsay) scores at 6, 12, 24, and 48 h. The secondary outcomes were rescue drug use rate, hemodynamic parameters (mean arterial pressure and heart rate), percutaneous oxygen saturation, respiratory rate, and occurrence of adverse reactions.

**Results:**

Group P exhibited lower resting and coughing VAS scores at 6, 12, 24, and 48 h, plus decreased incidence of nausea and vomiting within 48 h post-surgery compared with Group C (*p* < 0.05). No significant differences were observed in pruritus, sedation (Ramsay) scores, mean arterial pressure, heart rate, oxygen saturation, or respiratory rate between the two groups (*p* > 0.05).

**Discussion:**

The combination of sufentanil and promethazine for postoperative intravenous analgesia could effectively reduce adverse effects such as nausea and vomiting, contributing to postoperative pain relief.

## 1 Introduction

Enhancing postoperative analgesia is a key element of enhanced recovery after surgery (ERAS). It mitigates postoperative stress and immunosuppression, promoting rapid recovery (1). Thoracic surgeries often lead to significant pain due to injury receptors, surgical incisions, and thoracic drainage, which can result in complications such as tachycardia, arrhythmias, and pneumonia (2). Postoperative nausea and vomiting (PONV) represent prevalent postsurgical complications with an incidence of 20–30%, which can further complicate recovery, making improved analgesic protocols essential (3).

Promethazine, a derivative of phenothiazine, acts as a competitive H1 receptor antagonist while concurrently inhibiting dopaminergic and cholinergic receptors within the central trigger zone (CTZ). This dual mechanism diminishes nausea and vomiting through its pronounced antiemetic properties (4). Promethazine has also shown efficacy in inhibiting pain pathways and enhancing the analgesic effects of opioids, thereby reducing opioid requirements during the perioperative phase (5). The objective of this prospective, randomized, controlled, double-blind clinical trial was to evaluate the efficacy and safety of promethazine combined with sufentanil for postoperative analgesia in patients undergoing thoracic surgery, providing a scientific basis for pain management strategies.

## 2 Methods

### 2.1 Research agreements

This trial was conducted in compliance with the Declaration of Helsinki and approved by the Clinical Ethics Committee of Yongchuan Hospital, Chongqing Medical University. It was registered with the China Clinical Trials Center (registration number: ChiCTR2100044486). Written informed consent was obtained from all enrolled patients prior to their participation in the study.

### 2.2 Case selection

Sixty patients from the Department of Thoracic Surgery at Yongchuan Hospital, Chongqing Medical University, met the inclusion criteria for the study, which were defined as follows: (1) communicative capability; (2) undergoing elective thoracoscopic pneumonectomy under general anesthesia, including wedge resection, lobar resection, or segmental resection; (3) certified American Society of Anesthesiologists status I–III; (4) aged between 18 and 75 years; and (5) awake and extubated status.

The exclusion criteria comprised: (1) allergies to promethazine or any study drugs administered during this investigation; (2) usage of antiemetics within 48 h before the surgical procedure; (3) prolonged intake of opioids, sedatives, or non-steroidal anti-inflammatory drugs; (4) presence of endocrine abnormalities; (5) cognitive impairments; (6) cardiac conduction anomalies or arrhythmias; (7) additional cognitive impairments; and (8) conditions requiring postoperative intensive care unit (ICU) admission or involving communication difficulties. Researchers furnished comprehensive explanations regarding the visual analog scale (VAS) and analgesic pumps to the participating patients.

### 2.3 Randomization and blinding method

The participants in this study were grouped using a random chart method. The test group (Group P) received a combination of sufentanil (3 μg/kg), promethazine (1 mg/kg), and 0.9% sodium chloride solution (total volume of 100 mL). The control group (Group C) received sufentanil (3 μg/kg) and 0.9% sodium chloride solution (total volume of 100 mL). Patient-controlled intravenous analgesia (PCIA) was set at 1 mL per dose, with a lockout interval of 15 min.

To mitigate potential confounding factors, all thoracoscopic procedures were performed by the same team of thoracic surgeons. Staff members responsible for preparing the analgesic pumps for researchers were not involved in the study.

### 2.4 Methods of anesthesia

Upon patient admission to the operating room, intravenous access was established; routine monitoring of electrocardiogram (ECG), blood pressure, and oxygen saturation commenced. General anesthesia was performed with intravenous midazolam (0.05 mg/kg), penehyclidine hydrochloride (0.01 mg/kg), sufentanil (0.4–0.5 μg/kg), propofol (2–2.5 mg/kg), rocuronium bromide (0.6 mg/kg), and dexamethasone (8 mg). This was followed by fiberoptic bronchoscopy-guided tracheal intubation and initiation of mechanical ventilation. Anesthesia machine parameters were set as follows: tidal volume (VT), 8–10 mL/kg; respiratory rate (RR), 10–12 times/min; inspiratory-to-expiratory ratio, 1:2; and maintenance end-tidal carbon dioxide (PETCO_2_) level, 35–45 mmHg. Maintenance anesthesia included inhalation of 1–3% sevoflurane, continuous infusion of remifentanil at 0.1–0.2 μg/(kg.min) and continuous infusion of propofol at 0.05–0.1 mg/(kg.min), with additional single doses of rocuronium bromide (0.04 mg/kg) administered as needed. Ondansetron (4 mg) was given intravenously while the surgeon sutured the last layer of skin. Upon the patient regaining consciousness, extubation was performed, followed by the commencement of PCIA with a back infusion dose of 5 mL, a dose of 1 mL per administration, and a lock time of 15 min. The analgesic pump in Group P included sufentanil (3 μg/kg) + promethazine (1 mg/kg) + 0.9% sodium chloride solution (100 mL total), whereas the analgesic pump in Group C comprised sufentanil (3 μg/kg) + 0.9% sodium chloride solution (100 mL total).

The patient’s remedial analgesic on return to the ward was tramadol and remedial antiemetic was metoclopramide.

### 2.5 Outcome measures

The primary endpoints for this study were pain and sedation scores, which were recorded at 6, 12, 24, and 48 h following the surgical procedure. The secondary outcome measures were the rate of rescue drug usage, hemodynamic parameters, transcutaneous oxygen saturation, RR, and any adverse events observed. Hemodynamic parameters, including mean arterial pressure (MAP) and heart rate (HR); transcutaneous oxygen saturation; and RR, were documented upon the patient’s admission to the operating room, as well as at 6, 12, 24, and 48 h post-initiation of the analgesic pump. Pain and sedation scores were assessed at corresponding intervals postoperatively. The VAS was employed to assess postoperative pain levels both at rest and during movement, while Ramsay scores were utilized to gage sedation. The frequency of rescue drug use (tramadol) was monitored at 24 and 48 h post-surgery. Adverse events such as nausea and vomiting; respiratory depression; and pruritus were meticulously recorded throughout the observation period.

### 2.6 Sample size calculation

We calculated the sample size based on the primary outcome measure of VAS pain scores at different postoperative time intervals using a paired design. We assumed a clinically significant difference in VAS pain scores of 1.5 points (6), with a standard deviation of differences of 2.0. Aiming for a statistical power of 90% and a significance level of 0.05, the required sample size was calculated using PASS software. The results indicated that approximately 21 pairs of participants were needed per group. To account for potential dropouts, we increased the sample size by 20%, resulting in 27 pairs per group, making the total sample size 54 participants.

### 2.7 Statistical analysis

SPSS Statistics version 26.0 (IBM, Armonk, NY) was employed for statistical analysis. Measurement data are presented as mean ± standard deviation. Intergroup measurement data were compared using ANOVA, while data at different timepoints were analyzed using repeated-measures ANOVA. Adverse reactions are expressed as percentages and analyzed using the chi-squared test, with *p* < 0.05 considered statistically significant.

## 3 Results

### 3.1 General information

**Figure 1:**
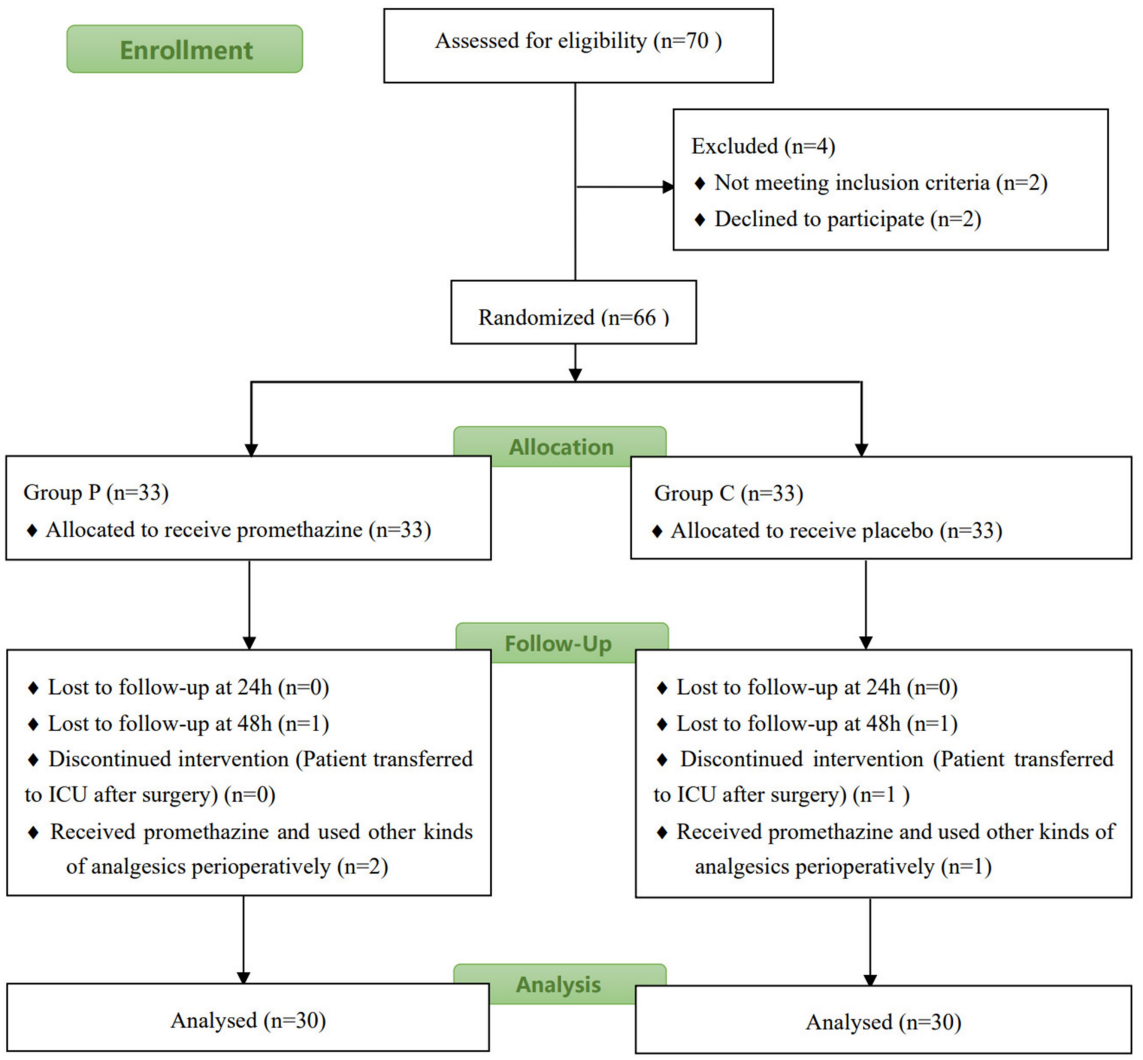
Flow diagram of patient recruitment.

Figure 1 shows the flowchart of the study. Of the 65 patients assessed for eligibility, 62 were enrolled and randomly assigned to the two groups. Two patients (one from Group P and one from Group C) were excluded from the study because they were admitted to the ICU for treatment post-surgery. The patients were recruited during May and June 2021. Statistical analysis revealed no significant differences in age, height, bodyweight, surgical time, sufentanil consumption during surgery, or type of operation between the two groups (*p* > 0.05, Table 1).

**Table 1:** Clinical characteristics and surgical characteristics of patients (x̄ ± s).

	Group P (*n* = 30)	Group C (*n* = 30)	*p*-value
Age (y)	59.20 ± 8.48	56.73 ± 7.44	0.236
Height (cm)	163.13 ± 4.44	162.76 ± 4.43	0.750
Body weight (kg)	60.00 ± 9.45	61.53 ± 9.35	0.530
Surgery time (min)	241.83 ± 47.39	253.17 ± 30.47	0.276
Consumption of sufentanil in surgery (μg)	35.53 ± 4.20	36.20 ± 3.47	0.505
Types of operation			
Wedge resection	8	13	
Segmental resection	10	7	
Lobectomy	12	10	
Apfel score for the risk of PONV			
0–1	9	11	
2	11	12	
≥ 3	10	7	

Clinical characteristics of patients had no significant difference between the groups. The Apfel risk score is based on four predictors: female sex, history of PONV and or motion sickness, smoking status, and postoperative opioid use. Each one of them each risk factor points, total score for the final Apfel score. Numerical values are given as the means ± SD.*Statistically significant (*p <* 0.05).

### 3.2 Comparison of VAS and Ramsay scores at different postoperative timepoints between groups

**Figure 2:**
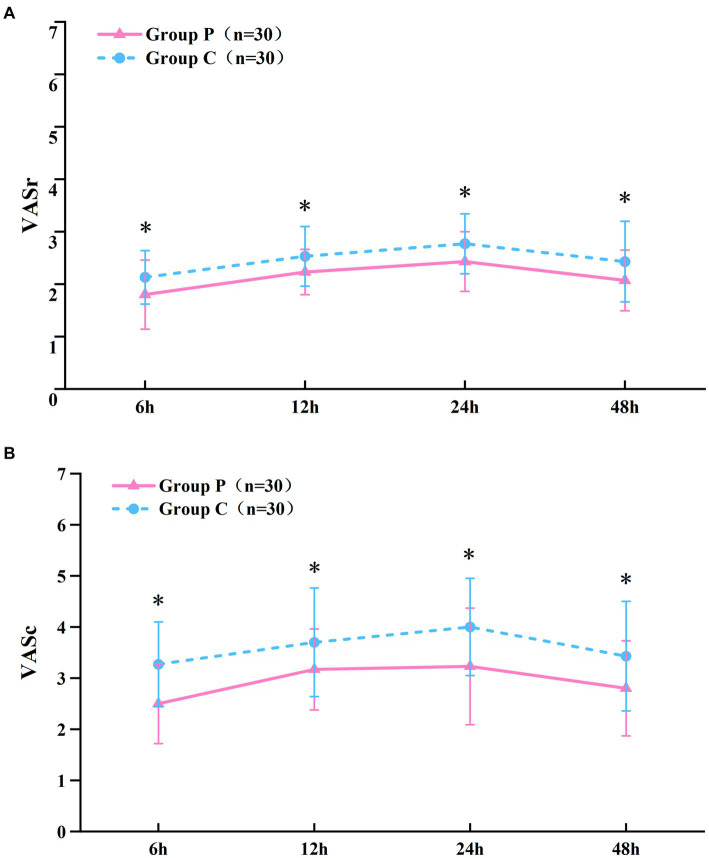
VAS scoring at rest **(A)**, at coughing **(B)**. Data given as mean and 95% CI of the mean. *P < 0.05: comparison between the Group P and Group C.

**Figure 3:**
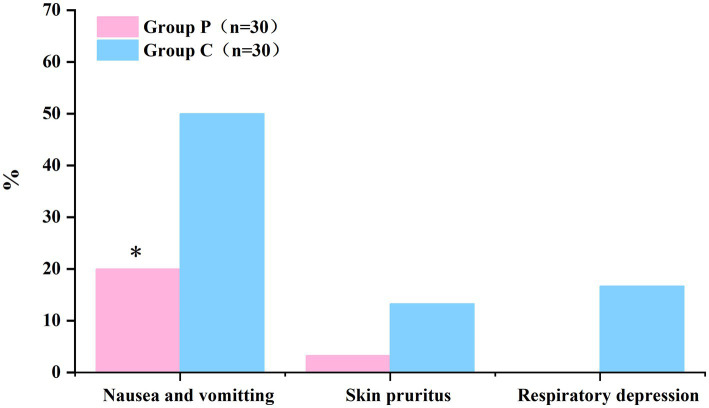
Ramsay sedation scores have no statistically differences between the Group P and Group C. Data given as mean and 95% CI of mean.

**Figure 4:**
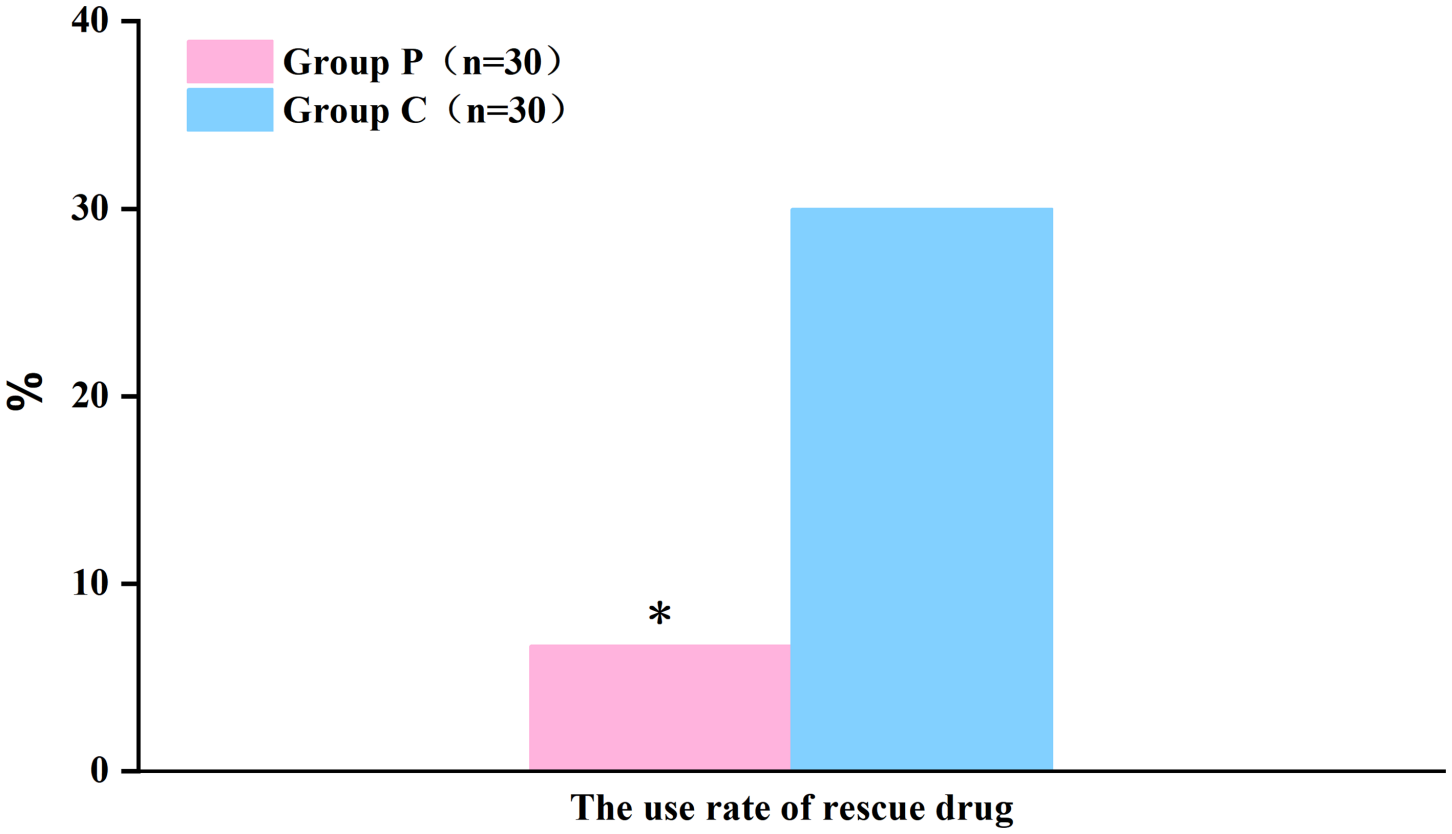
The use rate of rescue drug (Tramadol) within 48 h after surgery showed a significant difference between the groups. *P < 0.05: comparison between the Group P and Group C.

The resting (Figure 2A) and coughing (Figure 2B) VAS scores at 6, 12, 24, and 48 h postoperatively were significantly different (*p* < 0.05, Table 2), which suggested that promethazine alleviated pain during incision, indicating a better analgesic effect. However, no significant differences were observed in the Ramsay scores (Figure 3) at each timepoint between the two groups (*p* > 0.05, Table 2). The usage frequency of rescue drugs (tramadol) (Figure 4) in Groups P and C was 2 and 9, respectively, indicating a significant difference (*p* < 0.05, Table 3).

**Table 2:** VAS and Ramsay scores within 48 h postoperatively (x ± s).

Variable	Time	Group P (*n* = 30)	Group C (*n* = 30)	*P*-value
Rest VAS	6	1.80 ± 0.66*	2.13 ± 0.51	0.033
	12	2.23 ± 0.43*	2.53 ± 0.57	0.025
	24	2.43 ± 0.57*	2.77 ± 0.57	0.027
	48	2.07 ± 0.58*	2.43 ± 0.77	0.043
Cough VAS	6	2.50 ± 0.78*	3.27 ± 0.83	<0.001
	12	3.17 ± 0.79*	3.70 ± 1.06	0.031
	24	3.23 ± 1.14*	4.00 ± 0.95	0.006
	48	2.80 ± 0.93*	3.43 ± 1.07	0.017
Ramsay	6	2.23 ± 0.43	2.03 ± 0.41	0.072
	12	2.17 ± 0.38	2.10 ± 0.31	0.456
	24	2.00 ± 0.37	2.03 ± 0.18	0.661
	48	2.07 ± 0.25	2.03 ± 0.18	0.561

Rest VAS Score: refers to the VAS pain score when the patient is lying in bed at rest. Cough VAS Score: Refers to the VAS pain score throughout the process when the patient takes a deep breath and then coughs. Numerical values are given as the means ± SD.*Statistically significant (*p* < 0.05).

**Table 3:** Comparison of the use rate of rescue drug (Tramadol) within 48 h after surgery.

	Group P (*n* = 30)	Group C (*n* = 30)	*p*-value
The use rate of rescue drug (Tramadol)	2 (6.7%)*	9 (30%)	0.020

The use rate of rescue drug (Tramadol) within 48 h after surgery differed significantly between the groups.*Statistically significant (*p <* 0.05).

### 3.3 Comparison of clinical indicators between groups

No significant differences were observed in hemodynamic parameters (MAP and HR), percutaneous oxygen saturation, or RR between the two groups at the initial assessment when patients entered the operating room (T0) and at 6, 12, 24, and 48 h post-surgery (*p* > 0.05, Table 4).

**Table 4:** Comparison of clinical indicators between the two groups of patients.

	T_0_	6 h	12 h	24 h	48 h
MAP (mmHg)					
Group P	81.43 ± 7.01	83.13 ± 7.35	84.47 ± 7.22	83.97 ± 7.76	79.60 ± 6.47
Group C	81.07 ± 6.35	82.90 ± 6.74	84.73 ± 6.779	82.83 ± 7.97	80.27 ± 5.23
SPO_**2**_ (%)					
Group P	98.10 ± 1.16	98.13 ± 0.97	98.03 ± 1.07	97.83 ± 1.46	98.23 ± 0.94
Group C	97.73 ± 0.94	97.73 ± 0.94	97.63 ± 0.93	97.43 ± 1.14	97.80 ± 0.81
HR (Beats per min)					
Group P	95.03 ± 7.90	93.03 ± 10.57	94.90 ± 11.31	100.77 ± 13.28	94.30 ± 9.85
Group C	96.43 ± 6.98	94.47 ± 10.08	94.00 ± 9.17	99.37 ± 12.54	95.27 ± 8.51
RR (Breaths per min)					
Group P	22.57 ± 4.09	22.87 ± 4.70	24.17 ± 5.27	24.26 ± 6.09	23.80 ± 4.44
Group C	21.50 ± 2.86	21.63 ± 3.77	23.33 ± 4.95	23.53 ± 5.03	23.57 ± 3.56

Changes of MAP, SPO_**2**_, HR, RR. MAP, mean arterial pressure; SPO_**2**_, percutaneous oxygen saturation; HR, heart rate; RR, respiratory rate.

### 3.4 Comparison of adverse reactions within 48 h after surgery between groups

**Figure 5:**
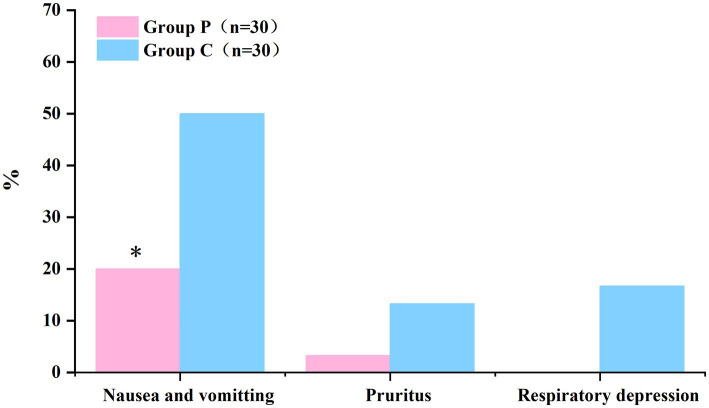
The incidence of nausea and vomiting within 48 h postoperatively was significantly lower in Group P than in Group C. (*p < 0.05).

The comparison of adverse reactions within 48 h after surgery are shown in Figure 5. The incidence rates of nausea and vomiting differed significantly between Groups P and C (20 and 50%, respectively; *p* < 0.05; Table 5). In patients with postoperative nausea and vomiting, we defined clinically significant PONV as two or more episodes of postoperative vomiting or retching that consistently affected daily life (these patients were given metoclopramide as rescue antiemetic). No significant differences were observed in the incidence rates of pruritus and respiratory depression within 48 h post-surgery between the groups (*p* > 0.05, Table 5).

**Table 5:** The incidence of adverse reactions within 48 h after surgery.

	Group P (*n* = 30)	Group C (*n* = 30)	*p*-value
Nausea and vomiting	6 (20%)*	15 (50%)	0.015
Participants with rescue antiemetic medication	1*	5	
Pruritus	1 (3.3%)	4 (13.3%)	0.353
Respiratory depression	0 (0%)	5 (16.7%)	0.052

The incidence of nausea and vomiting within 48 h differed significantly between the groups.*Statistically significant (*p <* 0.05).

## 4 Discussion

This study demonstrated that PCIA with a combination of sufentanil and promethazine post-thoracic surgery effectively reduced VAS pain scores and the incidence of nausea and vomiting without clinically relevant adverse effects such as hemodynamic disturbances or respiratory depression.

ERAS refers to a set of evidence-based perioperative optimization strategies, including refined surgical approaches, minimally invasive techniques; meticulous tissue handling; reduction of intraoperative trauma and hemorrhage; and shortening of operative durations, which are all aimed to effectively diminish the level of postoperative physiological and psychological stress among patients (7). Regarding thoracic surgery, the widespread use of videothoracoscopic surgery allows for minimally invasive surgical procedures that reduce surgical pain and stress, thereby reducing the frequency of use of thoracic segmental epidural analgesia, once the gold standard of thoracic postoperative analgesia. The widespread use of ultrasound technology has also allowed the use of various forms of nerve block techniques for postoperative analgesia following thoracic surgery. Visser et al. (8) conducted a comprehensive meta-analysis evaluating the benefits of systemic and regional analgesia following thoracic surgical procedures, involving 891 patients from 10 studies who underwent esophagectomy. Pain scores and pulmonary complications did not differ between the systemic and epidural analgesia groups at 24 and 48 h postoperatively (*p* > 0.05) (9). PCIA remains the most widely-used and effective method of postoperative analgesia due to its significant effects, rapid onset, absence of analgesic insensitivity, minimal risk of neural impairment, and timely control of pain onset with shock dosage (10).

Sufentanil, widely used as a postoperative analgesic, stands out for its rapid onset, immediate analgesic effects, and prolonged efficacy compared with conventional postoperative analgesics such as tramadol, dizocin, and aminotriol ketoacetic acid (11). However, being a potent opioid with high selectivity for μ-receptors, sufentanil is associated with various adverse effects (12). The μ-receptor sites, predominantly situated within the medial thalamus, ventricular system, and solitary tract nucleus, play a pivotal role in pain perception and integration. Specifically, the localization of *μ* receptors within the medial thalamus and ventricles is intricately linked to pain modulation, thereby endowing sufentanil with its pronounced analgesic efficacy (13). Nonetheless, given the proximity and integration of the solitary tract nucleus with the respiratory centers, the administration of sufentanil can precipitate significant adverse outcomes, notably postoperative respiratory depression (14, 15). The use of sufentanil for postoperative analgesia is associated with adverse effects, including nausea, vomiting, and pruritus, further highlighting the need for cautious application in clinical settings. Opioids exert their effects not only by activating the CTZ but also by stimulating the synthesis of a range of neurotransmitters, such as dopamine, acetylcholine, histamine, and pentagastrin (16). These biochemical interactions are pivotal in the pathogenesis of PONV and impair the efficacy of postoperative cough mechanisms in patients undergoing thoracic surgery, thereby prolonging the recovery process and affecting patient comfort (17). Consequently, the utilization of high opioid dosages is clinically recommended to be avoided for postoperative pain management. Therefore, we switched to drugs that could enhance analgesic efficacy while mitigating adverse reactions (18).

Promethazine, a phenothiazine antagonizing histamine H1 receptors, exhibits a multifaceted pharmacological profile, manifesting antihistaminic, anticholinergic, and notably central nervous system depressant properties (19). Although its therapeutic applications extend across a range of conditions, including cutaneous allergic reactions, vertigo, emesis, and as a complementary agent in analgesic regimens, its role in providing analgesia alone is debated. Research has indicated that H1 receptor antagonists, including promethazine, elicit notable analgesic effects in preclinical animal models (20). In a study exploring the contribution of histamine H1 receptors to hyperalgesia in rodent models, the administration of a specific H1 receptor agonist (for instance, FMPH) in conjunction with an H1 receptor antagonist was found to partially, yet significantly, mitigate the analgesic efficacy of the antagonist. This implies competition between the agonist and antagonist for the occupancy of the same receptor sites, suggesting that promethazine, an H1 receptor antagonist, mitigates the hyperalgesic responses triggered by H1 receptor activation (21). In another study utilizing a formalin-induced pain model, intrathecal administration of a selective H1 receptor blocker not only negated capsaicin-induced pain, but also diminished Fos protein expression within the dorsal horn neurons of the spinal cord, specifically in layers I and II. This intervention enhanced the antinociceptive effects of morphine, a phenomenon that was notably accentuated in H1 receptor-deficient mice (22). These findings support integrating promethazine into pharmacotherapeutic regimens to manage pain-related conditions, particularly those characterized by histamine-driven pain pathways. In this experiment, we evaluated the analgesic effect by observing the resting and coughing VAS scores of patients during normal breathing and coughing at different times after surgery. In patients undergoing thoracic surgery, appropriate postoperative coughing contributes to lung recuperation and pulmonary function recovery. Therefore, we designed wound pain scores in the resting and coughing states, which are also more conducive to evaluating patients’ early postoperative recovery. In this study, the experimental group exhibited significantly lower resting VAS and cough pain scores than the control group at 6, 12, 24, and 48 h, without discernible differences in Ramsay sedation scores between the groups. This implies that promethazine’s analgesic effect is not attributable to a sedative and pharmacological action. The combination of promethazine and opioids demonstrated an opioid-sparing effect, significantly reducing the total opioid usage and duration of postoperative stay in the post-anesthesia care unit (23).

Recent studies have demonstrated that histamine and several other mediators enhance capillary permeability, stimulate the neuronal release of substance P for nociceptive transmission, and induce mast cells to secrete additional histamine, which facilitates the entry of substances like bradykinin into the vasculature, exacerbating inflammation (24). Grundy et al. (25) found that histamine can regulate c-fiber activity by increasing c-fiber mechanosensitivity and activating silent C-fiber memory, affecting ion channels such as TRPV1, which in turn modulates C-fiber activity. The utilization of H1 receptor antagonists presents a viable strategy for mitigating the onset of peripheral inflammation and C-fiber pain sensitization through their pronounced anti-inflammatory properties, consequently diminishing postoperative pain intensity. Local and systemic inflammatory responses to surgical trauma are natural responses of the body to surgical injury. Postoperative infections, particularly poor incisional healing and pulmonary complications, such as pneumonia and pleural effusion are common in patients undergoing thoracic surgery (26). Despite the minimally invasive nature of thoracoscopic surgery, the persistence of inflammation can complicate pain management and potentially contribute to the risk of cancer recurrence (27, 28). Our findings indicated that the anti-inflammatory properties of promethazine were not conclusive. However, the premise that promethazine’s impact on inflammatory processes may enhance analgesic effectiveness warrants further investigation. This can be achieved by quantitatively assessing the concentrations of inflammatory markers in patients’ blood samples at various intervals post-surgery.

Joly et al. (29) found that clinically relevant concentrations of remifentanil enhanced the response of the N-methyl-D-aspartate (NMDA) receptor system and caused depolarization of the spinal dorsal horn neurons, engendering a dose-dependent escalation in nociceptive hypersensitivity (30). Concurrently, Liu et al. (31) reported that spinal protein kinase C plays a pivotal role in remifentanil-elicited nociceptive sensitization in rats through the modulation of phosphorylation levels of NMDA receptors incorporating the R1 subunit (32). Adolph et al. (33) demonstrated that promethazine attenuates NMDA-mediated membrane currents in a non-competitive, concentration-dependent fashion. This inhibitory effect provides a new idea for its application in analgesia, sedation, and nociceptive hypersensitivity inhibition. Therefore, integrating sufentanil with promethazine for PCIA not only amplifies sufentanil’s analgesic properties but also mitigates the nociceptive hypersensitivity induced by perioperative opioid administration, including remifentanil.

Promethazine selectively inhibits the emetic chemoreceptor zone within the medulla oblongata and/or directly diminishes the excitability of the vomiting center. Analysis of our dataset revealed a marked reduction in the incidence of nausea and vomiting among patients in the experimental group within 48 h post-surgery, in contrast to that in the control group. Additionally, we observed a higher incidence of respiratory depression among patients in the control group. Opioids such as sufentanil attenuate the response of the peripheral and central chemoreceptors to varying concentrations of carbon dioxide and oxygen, affecting respiratory rate and leading to respiratory depression (34). Clinically, respiratory depression would be expected to manifest as a slowed respiratory rate. However, a study on continuous postoperative respiratory monitoring indicated that bradypnea is a rare manifestation of respiratory depression (35). Another analysis of postoperative respiratory depression reported that the primary symptom preceding critical events is somnolence, accounting for 62% of cases (36). To prevent severe respiratory depression events, we included decreased respiratory rate (<8–10 breaths/min), pulse oximetry saturation (SpO_2_) <90%, and excessive somnolence in the definition of respiratory depression. Studies examining key respiratory events have shown that respiratory depression is often recorded as somnolence in nursing records (37). Therefore, to avoid potential adverse outcomes, we classified excessive somnolence as a manifestation of respiratory depression, which may have led to an increased number of recorded instances of respiratory depression. Although there was no statistically significant difference in the incidence of respiratory depression between the control and experimental groups, the absence of recorded respiratory depression in the experimental group could be attributed to several factors. First, the “wake-up effect,” where patients rendered somnolent by opioids may be briefly awakened during assessments, leading to inaccurate recording of respiratory depression. Second, patients may not have adhered to continuous vital signs monitoring post-surgery; previous studies have shown that routine monitoring often fails to detect most respiratory depression events after patients are transferred to regular wards (38). Third, the limited sample size may have statistically prevented the capture of such events. Meanwhile, the high incidence of respiratory depression suggests that we should reduce the dosage of sufentanil in analgesic pumps in future clinical applications and adopt multimodal analgesia strategies, such as regional nerve block techniques or the combination with non-opioid analgesics, to mitigate such adverse effects.

This trial has some limitations. This study primarily focused on the overall effect of combining promethazine with sufentanil for PCIA. However, the potential influence of sex and age on acute pain intensity should not be overlooked. Previous research indicates that female patients often report higher pain intensity, which may be related to physiological and psychological factors. Additionally, as age increases, pain perception and response can change; older patients may exhibit lower sensitivity to acute pain but have a higher incidence of chronic pain (39).Given that the primary aim of this study was to evaluate the effectiveness of promethazine combined with sufentanil for postoperative PCIA, and due to limitations in sample size, simplification of the study design, and the need to ensure data focus, we did not include these variables in the main analysis. Nevertheless, recognizing their possible impact on the results, future studies should consider expanding the sample size and conducting subgroup analyses of variables such as sex, age, and type of surgery. This would allow for a more detailed evaluation of their roles in postoperative pain management and help optimize personalized pain management strategies. According to previous research, most evaluations of postoperative analgesic efficacy in patients undergoing thoracoscopic surgery have recorded VAS scores at rest and during coughing but have not included VAS scores during forced deep breathing. Early postoperative deep breathing can improve lung function, reduce atelectasis, enhance oxygenation, and effectively prevent postoperative pulmonary complications. Therefore, future studies should record VAS pain scores during deep breathing after thoracoscopic surgery to more comprehensively evaluate analgesic efficacy. Patient-reported analgesic satisfaction was not recorded in our study, potentially influencing the comprehensive evaluation of analgesic effectiveness. However, based on the use of postoperative remedial analgesic, the analgesic effect in the experimental group was greater than that in the control group. We stratified the risk of PONV in all included patients and administered dexamethasone at the induction of anesthesia and ondansetron before the end of surgery as a prophylactic measure for PONV. Promethazine has been shown to prevent PONV. Although we classified postoperative PONV as an adverse event and recorded its incidence, we did not evaluate its severity using a specialized rating scale. We defined clinically significant PONV as two or more episodes of postoperative vomiting or retching that consistently affected daily life. Metoclopramide was actively administered as a remedial measure to all patients who experienced PONV; postoperative follow-up showed that the nausea and vomiting symptoms of the patients were significantly improved by remedial antiemetic measures. The dose of promethazine for PCIA was set at 1 mg/kg, and the results of the trial confirmed that it had good analgesic effects without significant sedative effects or side effects. However, the optimal dose of promethazine for PCIA is still unclear. Future research should explore a wider range of dosages to determine the most effective analgesic dose of promethazine for PCIA.

In conclusion, the combination of sufentanil and promethazine for postoperative analgesia post-thoracic surgery significantly reduced postoperative pain and associated side effects. Future studies are warranted to explore the underlying mechanisms of promethazine’s analgesic properties and assess its application time and dose during surgery, its impact on nociceptive sensitization, and its efficacy in postoperative analgesia.

## Data Availability

The raw data supporting the conclusions of this article will be made available by the authors, without undue reservation.
